# Feasibility of the Golf Recreational Exercise for Enhanced Survivorship in prostate cancer survivors undergoing hormone therapy

**DOI:** 10.3389/fonc.2026.1844481

**Published:** 2026-08-11

**Authors:** Guanrong Cai, Jacek Pinski, Lori A. Michener, E. Todd Schroeder, Daniel Kirages, Joy Tu, Kimiko Yamada, George Salem

**Affiliations:** 1 Division of Biokinesiology and Physical Therapy, University of Southern California, Los Angeles, CA, United States; 2 Keck School of Medicine, University of Southern California, Los Angeles, CA, United States

**Keywords:** exercise oncology, golf, hormone therapy, prostate cancer, recreational exercise, multimodal activity

## Abstract

**Background:**

Prostate cancer (PCa) survivors undergoing androgen deprivation therapy (ADT) experience significant physical and psychosocial side effects that negatively impact quality of life. Although exercise is effective in mitigating these effects, adherence to traditional programs remains low. The Golf Recreational Exercise for Enhanced Survivorship (GREENS) program was developed as a novel multimodal recreational activity (MRA) integrating structured exercise with golf to enhance engagement and sustainability.

**Objective:**

To evaluate the feasibility of the GREENS program in PCa survivors on hormone therapy, focusing on safety, adherence, enjoyment, and satisfaction, while exploring participant experiences through mixed methods.

**Methods:**

This single-arm, within-subjects feasibility study included a 5-week delayed-entry control period followed by a 10-week intervention. Fifteen participants were enrolled, with 11 initiating and 10 completing the intervention. Participants attended twice-weekly 90-minute group sessions combining exercise and golf activities. Feasibility outcomes included attendance, within-session adherence, adverse events (CTCAE), Physical Activity Enjoyment Scale (PACES-8), and a 4-item satisfaction scale. Post-intervention exit interviews were analyzed using thematic analysis.

**Results:**

Among completers, attendance was 90% and within-session adherence was 98.9%. No adverse events ≥ Grade 2 were reported, indicating high safety. Mean PACES-8 scores ranged from 52.9 to 54.8, and satisfaction scores ranged from 26.2 to 27.1 across program phases, demonstrating high enjoyment and satisfaction. All participants reported a strong willingness to recommend the program (mean = 7/7), and high likelihood of continued participation (mean = 6.4/7). Qualitative analysis identified four key themes: perceived value and satisfaction, program design and delivery, challenges and barriers, and expectations and reflections. Participants highlighted holistic health benefits, social support, skill development, and program structure as major facilitators of engagement; while scheduling and health-related factors were the primary barriers.

**Conclusion:**

The GREENS program is a safe, highly adherent, and well-accepted MRA for PCa survivors on hormone therapy. The integration of exercise with a socially engaging and skill-based activity such as golf may enhance motivation and long-term participation. These findings support further investigation of golf-based and other recreational interventions in exercise oncology.

## 1 Introduction

Prostate cancer (PCa) is the most common cancer diagnosis among men in the United States (1, 2). Survivors commonly undergo hormone treatment, including androgen deprivation therapy (ADT) and androgen receptor blockers (1, 3). Hormone treatment is associated with adverse effects such as fatigue, muscle loss, decreased bone density, stress, anxiety, and diminished quality of life (4–6). Exercise can mitigate many of these effects; however, adherence to traditional exercise programs remains a persistent challenge in this population due to lack of motivation, access, time, and perceived enjoyment (7–9).

To address these issues in exercise oncology and cancer survivorship care, the Golf Recreational Exercise for Enhanced Survivorship (GREENS) program was developed as a novel, multimodal, recreational activity (MRA) combining structured exercises with the psychosocial and cognitive benefits of recreational golf. Designed specifically for PCa survivors on hormone therapy, the GREENS program integrated evidence-based exercise principles within the structure of a group-based, supervised golf experience (10, 11). The GREENS program was built upon previously tested golf-based MRAs in older male military veterans and healthy older-adult males and females (12–16). Each PCa survivor’s journey and set of challenges are unique; the current study employs quantitative and qualitative methods to conduct in-depth analysis of each participant’s physical and psychosocial characteristics and their related subjective feedback about the program. This mixed methods approach was used to gain insight into the perceived facilitators and barriers to participation and engagement, and to better understand the benefits and challenges of both the program design and its implementation. These insights can inform the development of effective, creative, socially engaging, and sustainable MRAs, which can be tailored to cancer survivors.

The primary aim of the study was to evaluate the feasibility of the GREENS program across multiple dimensions, including safety, adherence, and acceptability (e.g., satisfaction and enjoyment) (17). We hypothesized that the program would be feasible with high: 1) safety (no above CTCAE grade 2 adverse events), 2) adherence (>80% attendance and >90% within-session activity adherence), 3) enjoyment (PACES-8 scale average score ≥ 50 and positive qualitative results), and 4) satisfaction (4-item satisfaction scale score ≥ 25 and positive qualitative results).

## 2 Methods

### 2.1 Study design

The study was a single-arm observational study, that used a within-subjects, repeated-measures design, with a 5-week delayed-entry control period, and a 10-week GREENS intervention period (18, 20, 35). The study integrated both quantitative and qualitative assessments to capture different aspects of feasibility, including safety, adherence, enjoyment, satisfaction, and participant experiences (18, 19). The study was approved by the University of Southern California (USC) Institutional Review Board (#HS-23-00366), Clinical Investigations Committee, and registered at ClinicalTrials.gov (ID: NCT06500169). Expanded details of the study design were previously published (13, 20).

### 2.2 Recruitment

Participants were recruited from USC-affiliated oncology clinics and through community outreach. Inclusion criteria included: (1) diagnosis of prostate cancer, (2) current or recent ADT use, (3) medical clearance for moderate physical activity, and (4) ability to commit to twice-weekly group sessions. Recruitment efforts focused on both referrals from the USC Norris Comprehensive Cancer Center’s Prostate Cancer clinic and identifying potential participants via USC i2b2 informatics data warehouse. Medical clearance forms and informed consents were obtained prior to enrollment.

### 2.3 Study execution

The research team consisted of a full-time Research Coordinator, a PGA professional golf instructor, Research Associates, and faculty advisors from USC. The Research Coordinator took charge of all study activities. The PGA professional was present at all training sessions to deliver golf skill instruction and direct on-course play. Research associates assisted with recruitment, exercise instructions, and on-field data collection. The study took place at Monterey Park Golf Course, Monterey Park, CA, a public golf course in Southern California. Environmental and logistical factors such as weather, course access, national holidays, and natural disasters were taken into consideration when scheduling data collection and training sessions. Sessions were postponed as needed to maintain participants’ safety and maximize attendance rate and program enjoyment.

Participants were divided into 3 groups of 5 and each group was scheduled to attend 20 training sessions in 10 weeks. Each 90-minute training session integrated a dynamic warm-up, golf skill instruction, course play, and structured physical activity components (e.g., walking, balance training, functional strength movements). Phase 1 of the training program occurred from Week-1 to Week-4. Here, participants spent 45 minutes on bodyweight and band-resisted exercises and 45 minutes on golf skill learning and practice. Phase 2 of the training program occurred from Week-5 to Week-7. During this period participants performed 30 minutes of warm-up exercises then spent the rest of the sessions on golf practice and transitioned to 1–3 holes of on-course golf play. Phase 3 of the program occurred from Week-8 to Week-10, where participants performed 15 minutes of warm-up exercises then proceeded to play 7 to 9 holes on-course (12, 13). The program was designed to promote moderate-intensity exertion while fostering social interaction and cognitive engagement through the sport of golf. Expanded details of the exercise and golf program were detailed in previously published methods paper (13, 20).

### 2.4 Feasibility outcome measures

*Adherence rate* was assessed through attendance records and session logs. *Activity adherence* was further evaluated by tracking minutes missed from prescribed activities during each session. Reasons for missed sessions and missed minutes were documented. The program was considered “adherent” if 80% attendance and 90% within-session adherence to the program activities were achieved. *Dropout rates* and reasons for attrition were recorded. *Safety* was monitored continuously, with all adverse events and physical complaints documented by study staff. Adverse events are reported according to the National Cancer Institute’s Common Terminology Criteria for Adverse Events (CTCAE) (21). The program was considered “safe” if there were no adverse events greater than CTCAE grade 2. Acceptability was assessed by *enjoyment* (Physical Activity Enjoyment Scale-8; PACES-8) and *satisfaction* (4-item scale) questionnaires, which are both 7-point Likert-scale instruments (22, 23). The PACES-8 scoring range is 8 to 56 and higher score indicates higher physical activity enjoyment. The 4-item satisfaction scale scoring range is 4 to 28 and higher score indicates higher satisfaction with the outcome of physical activity (23). These measures were used to evaluate the perceived value of the program and its components at the end of each training phase of the training program. The program was considered “enjoyable” if PACES-8 average scores were ≥ 50 at the end of all three program phases and that qualitative results identified positive themes of enjoyment (22, 24). Similarly, the program was considered “satisfactory” if the 4-item satisfaction scale average scores were ≥ 25 at the end of all three program phases and that qualitative results identified positive themes of satisfaction (22, 24).

### 2.5 Qualitative analysis

At the end of the 12-week program, participants completed an exit survey that captured reflections on the overall experience, perceived benefits, suggestions for improvement, and qualitative feedback on program structure, logistics, and content. Following Braun and Clarke’s six-phase approach, a thematic analysis was conducted. The interview script and analytical methods were previously published (20, 25, 26). In brief, the audio recordings of the interviews were transcribed verbatim to identify recurring phrases. After further reviews, these phrases were organized into subgroups and themes representing the participants’ opinions. This analysis was designed to provide nuanced details regarding the participants’ experiences in the program.

Additionally, there was a question regarding the participants’ willingness to recommend the program to their peers, which had a 7-point Likert-scale response where “1” meant “absolutely will not recommend” and “7” meant “totally will recommend”. Similarly, another question asked the participants’ likelihood to continue participation if there was a similar program, which also had a 7-point Likert-scale answer where “1” meant “unlikely at all” and “7” meant “very likely”. Average scores of 6 or above indicated a high willingness to recommend the program and the likelihood of continuing participation.

## 3 Results

**Figure 1:**
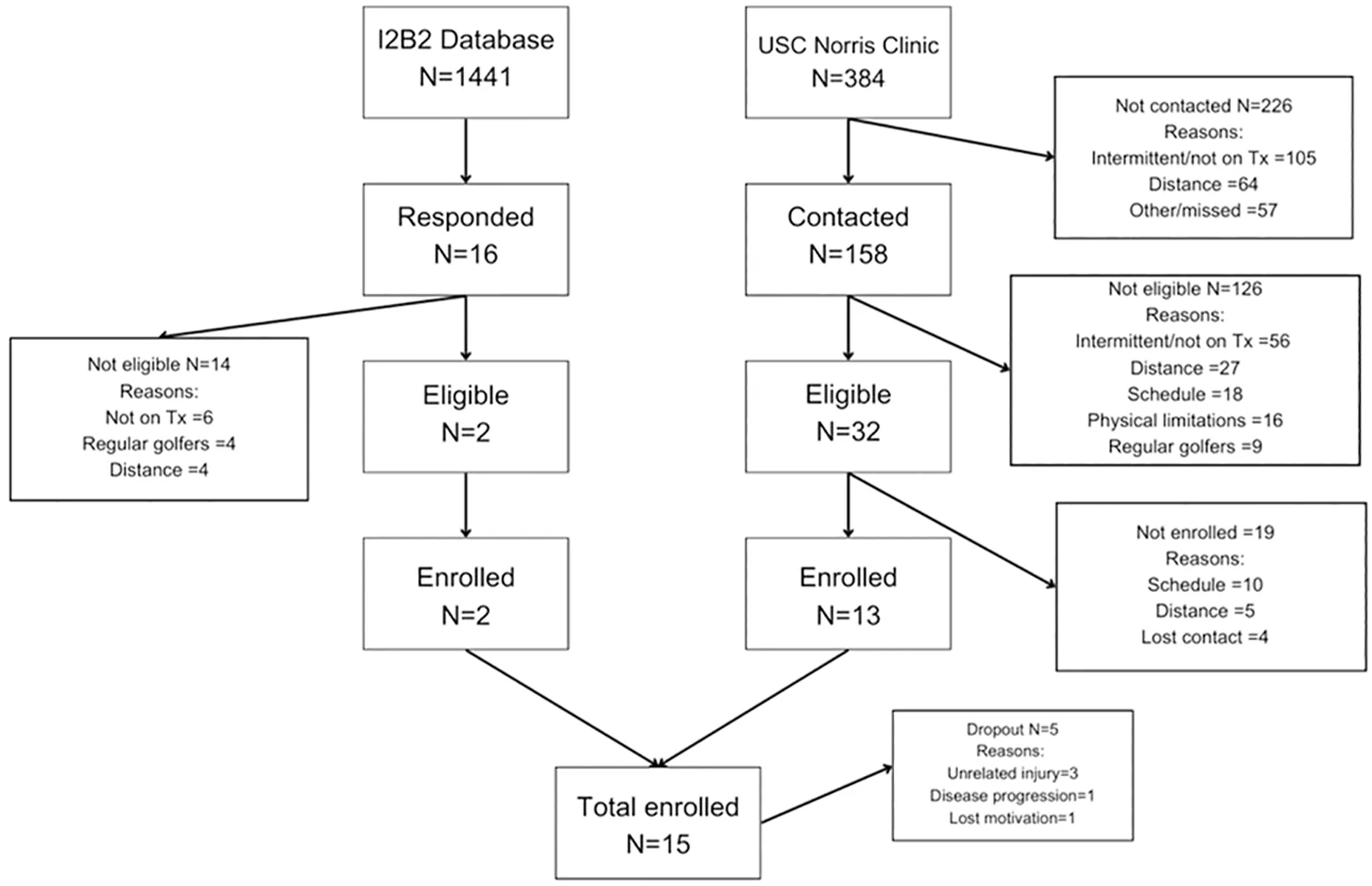
Recruitment and enrollment flowchart.

Fifteen participants enrolled in the study. Recruitment took place from January 2024 to February 2025 (**Figure 1**). All fifteen enrolled participants participated in the baseline assessment, but 4 participants discontinued the study during the 5-week control period due to unrelated injuries (n=2), disease progression (n=1), and loss of motivation (n=1). Of the 11 participants who started the intervention, 10 completed all study procedures. One participant discontinued after week-6 of the intervention due to worsening hip osteoarthritis symptoms. Participant demographics are listed in **Table 1**. For those who completed the study and intervention, training session attendance rate was 90% (180/200 sessions), and within-session activity adherence rate was 98.9% (15925/16110 minutes). The majority of the missing minutes were due to traffic, bathroom breaks, and requested rest breaks (**Table 2**). There were no Grade 2 or above adverse events according to the CTCAE. There was one episode of low blood pressure for a participant during the first training session; however, he recovered after resting for 5 minutes. Another participant underwent active radiation treatment during the intervention period and experienced increased fatigue in some of the training sessions. He took extended rest breaks and ended one session 10 minutes early.

**Table 1:** Participant demographics.

Demographics	Started training	Enrolled, did not start training
Participants number	N=11	N=4
Age (years)	73.5 ± 4.4	73.8 ± 9.2
Height (cm)	172.2 ± 7.5	182.0 ± 5.8
Mass (Kg)	77.8 ± 12	88.5 ± 16.8
Race & Ethnicity	White = 5 (2 Hispanic) Asian = 5 Black = 1	White = 2 Black =1 Native American/Black=1
Average time on hormone treatment (months)	26.8 ± 34.6	46.8 ± 35.9
Reasons for discontinuation	Hip osteoarthritis and back spasm = 1	Unrelated injuries/symptoms = 2 Disease progression = 1 Lost motivation = 1

**Table 2:** Adherence results.

Session attendance	180/200 (90%)
Missed-sessions reasons	Work/business: 6 Family: 5 Medical: 4 Unrelated injury/sickness: 3 Other: 2 (LA fire, holiday travel)
Session activity adherence	15925/16110 (98.9%) minutes
Missed-activity reasons	Traffic Bathroom breaks Extended rest breaks

**Figure 2:**
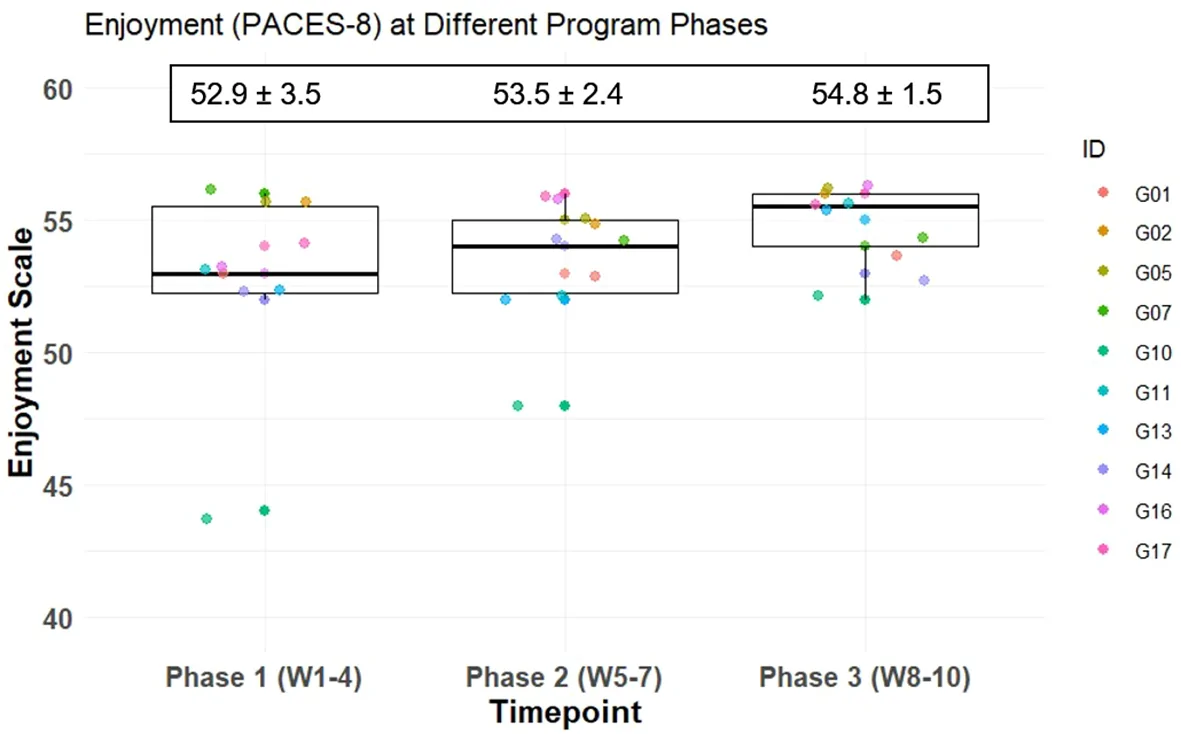
Enjoyment (PACES-8) scores at different program phases.

**Figure 3:**
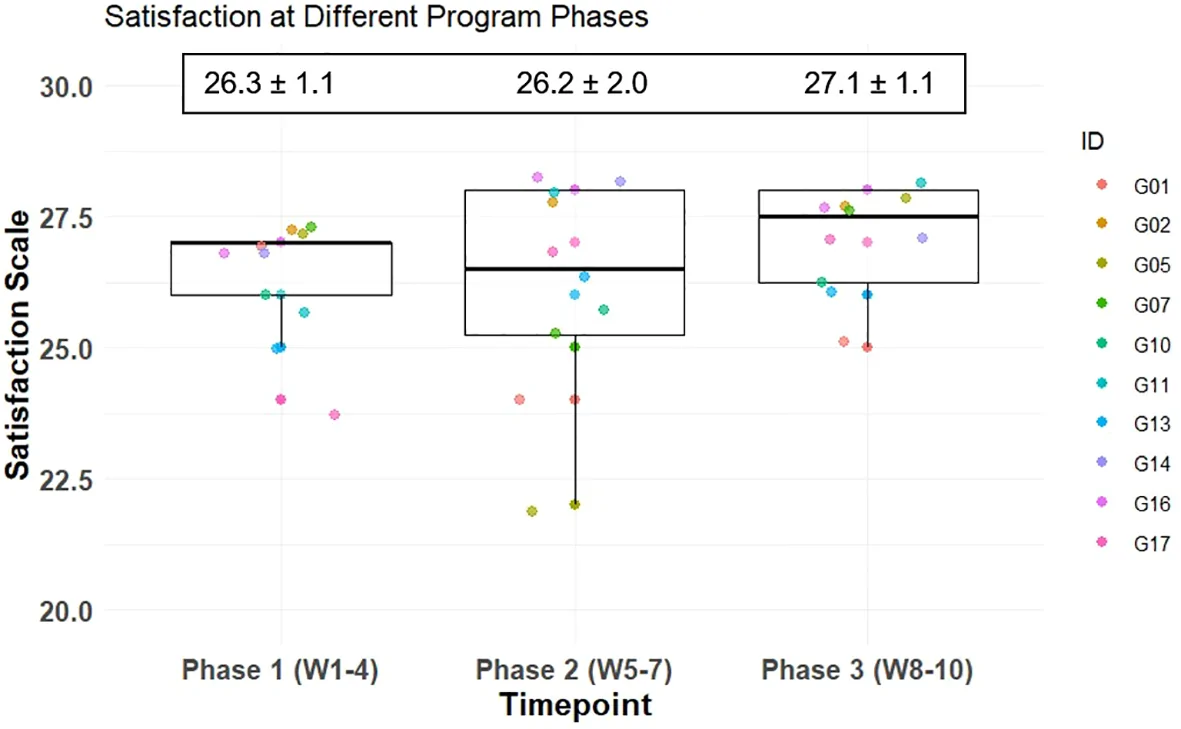
Satisfaction scale (4-item) scores at different program phases.

In terms of subjective enjoyment, the PACES-8 questionnaire scores were 52.9 ± 3.5, 53.5 ± 2.4, and 54.8 ± 1.5, respectively for the 3 training program phases (highest possible score is 56) (**Figure 2**). Similarly, the 4-item satisfaction scores were 26.3 ± 1.1, 26.2 ± 2.0, and 27.1 ± 1.1, respectively for the 3 training program phases (highest score possible score is 28) (**Figure 3**).

### 3.1 Qualitative results

**Figure 4:**
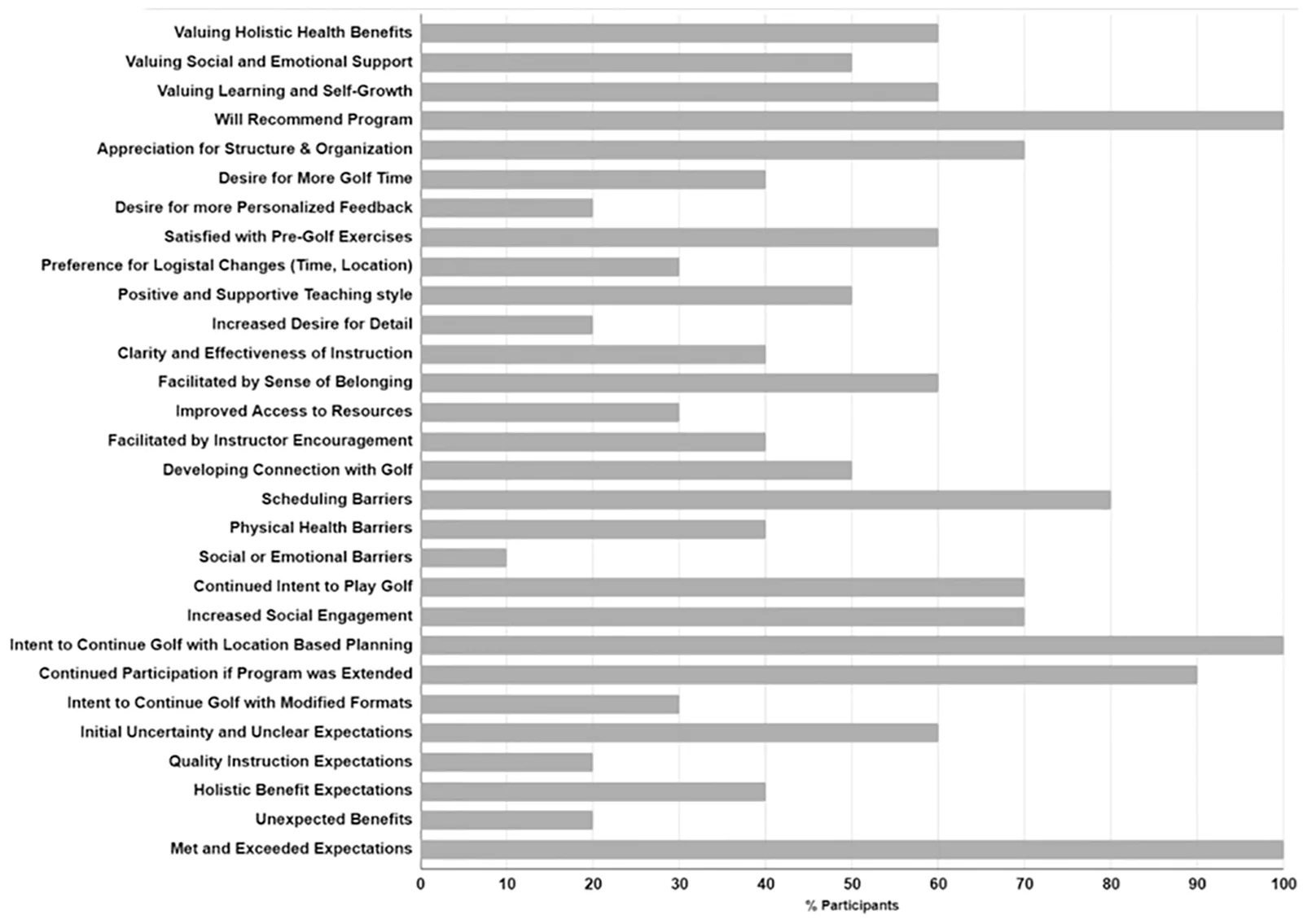
Qualitative results: percentage of participants represented in each category.

The thematic analysis identified 29 categories (identified in italics), organized into 4 broad themes: Perceived Value & Satisfaction, Program Design & Delivery, Challenges & Barriers, and Expectations & Reflections (**Supplementary Table 3**). Each theme included specific subgroups: Overall Satisfaction and Structure & Format under Perceived Value & Satisfaction; Instruction Quality and Engagement & Motivation under Program Design & Delivery; Time & Scheduling, Physical Health Barriers, and Social Barriers under Challenges & Barriers; and Continued Engagement and Program Expectations under Expectations & Reflections. Participant quotations, accompanied by study ID, are provided to give depth to the findings. Participants frequently used the names “You and Kevin” to refer to the research coordinator and the PGA professional instructor, Kevin Norwall. Percentage of participants represented in each category is illustrated in **Figure 4**.

The average score for the question regarding the willingness to recommend the program to others was 7 (± 0), which represented the highest score “totally will recommend”. All participants indicated that they “totally will recommend” the program. The average score for the question regarding the likelihood of continuing participation if such a program exists long-term was 6.4 (± 1.5), out of the highest score of 7 which represents “very likely”. Only one participant indicated that he would be “unlikely” to continue participation at this frequency. Reasons for their scores are discussed in the following sections.

#### 3.1.1 Theme 1: perceived value & satisfaction

##### 3.1.1.1 Subgroup #1: overall satisfaction

Six participants noted the value in the program’s *holistic health benefits*, noting the improvement in physical ability, mental ability, and overall outlook on life. Several described how participating in golf kept them active in ways that went beyond structured exercise, helping them regain strength, flexibility, and coordination while also providing mental stimulation. Participants noted the multidimensional benefit of the program, including physical health, cognitive health, and emotional well-being, supporting overall satisfaction and motivated participation.

Valuing *social and emotional support* was also important to the participants’ overall satisfaction with the program. Five participants described the program as more than just a sport, emphasizing its role in building a community. Many participants noted that interacting with peers who had similar experiences created solidarity and emotional support. The camaraderie, encouragement, and sense of belonging from both peers and instructors made participation both meaningful and motivating.

Six participants emphasized that the program’s focus on *learning and self-growth* made participation sustainable. Structured instruction from professionals provided clarity and guidance in a supportive environment that made complex skills accessible. Participants valued the sense of accomplishment that came from learning new skills, challenging themselves, and building self-efficacy. This combination of structured learning, personal growth, and social interaction made the program worthwhile among the participants.

Participants’ willingness to *recommend the program* indicates strong perceived value and acceptability. All ten participants that completed the golf program reported they would encourage others to join, often citing the program’s holistic benefits. High recommendation rates suggest that participants found the program feasible to participate in over time.

In essence, these reflections show that participants not only valued the program’s combination of physical activity, social support, personal growth, and structured instruction but also experienced high overall satisfaction.

##### 3.1.1.2 Subgroup #2: structure and format

Seven participants expressed strong *appreciation for the structure and organization* of the program. Many highlighted the thoughtful sequencing of exercises, driving range sessions, and time on the course contributed to an engaging and manageable experience. Clear scheduling, punctuality, and the provision of equipment were frequently cited as factors that enhanced both feasibility and satisfaction.

While participants appreciated the structure, four participants suggested a *desire for more golf time* to increase movement and engagement. Participants commented on the fun and challenge of golf intensified in the latter portion of the program, suggesting that extending on-course time could further enhance the experience.

Two participants expressed interest in more *personalized feedback* from instructors. Others mentioned a desire for guidance on club selection or technique while on the course, which could further optimize engagement and confidence.

*Satisfaction with the pre-golf exercises* was also expressed, with six participants noting their relevance and effectiveness in preparing for play. A few suggested minor adjustments to the location or integration with the driving range to improve efficiency and inclusion.

Finally, three participants highlighted *preferences for logistical changes*, including session timing, course variety, and group size. Some suggested earlier start times or occasional alternative course locations to diversify the experience. Participants also commented on expanding group size to enhance social dynamics, allowing more opportunities to form peer connections.

Overall, the feedback indicated that the program’s well-planned structure, combination of exercises and golf, and supportive instruction contributed to a strong sense of satisfaction, while minor adjustments could further enhance engagement and overall participant experience.

#### 3.1.2 Theme 2: program design & delivery

##### 3.1.2.1 Subgroup #1: instruction quality

Six participants consistently praised the instructors, highlighting a *positive and supportive teaching style* as central to their satisfaction with the program. They described the instructors as encouraging, approachable, and attentive, creating a low-pressure environment that made learning accessible and enjoyable. Participants emphasized that this supportive approach contributed to their confidence and engagement throughout the program.

Two participants expressed a *desire for increased detail* in instruction, particularly regarding technical aspects of golf. They noted that while the fundamentals were covered, additional guidance on grip, stance, and angulation would have enhanced their learning experience.

The *clarity and effectiveness of instruction* were also highlighted by four participants, who appreciated concise, actionable cues that allowed them to make immediate improvements. These instructional qualities were seen as critical for participant learning and confidence, reinforcing the feasibility of continuing the program.

##### 3.1.2.2 Subgroup #2: engagement and motivation

Many reported that the program fostered a strong *sense of belonging*, with six participants emphasizing the value of connecting with peers and feeling supported in a welcoming environment. These reflections highlight how support and camaraderie within the group created a space where participation felt enjoyable.

*Improved access to resources* also played a role in motivation for three participants. Access to equipment, lessons, and golf sessions enhanced engagement and reduced barriers to participation. These resources made participation both easier and more motivating.

*Instructor involvement* further facilitated motivation for four participants. Participants appreciated encouragement, guidance, and support from program leaders. “You [the research coordinator] and Kevin” were frequently cited as key to maintaining engagement, illustrating how program leader support and engagement sustained commitment to the program.

Finally, six participants described developing a *connection with golf*, which increased both enjoyment and adherence. Several noted rediscovering or improving skills they had not practiced for years, while others expressed excitement about returning to play in the future, signaling ongoing engagement. For many, engagement in golf has become more than an activity, but a source of continuity.

These reflections demonstrate that by fostering belonging, promoting holistic health, removing barriers through resources, providing supportive instruction, and nurturing a renewed connection with golf, the program offered participants a meaningful, multidimensional pathway to both engagement and sustained participation.

#### 3.1.3 Theme 3: challenges and barriers

Although participants encountered a variety of barriers related to time, health, and social responsibilities, these challenges were often framed less as deterrents and more as tests of commitment. In many ways, the effort to navigate around these barriers underscored the value participants placed on the program and their sense of accountability both to themselves and to the group.

##### 3.1.3.1 Subgroup #1: time and scheduling

*Timing and scheduling* emerged as the most frequently mentioned challenge by eight participants, particularly in balancing medical appointments, work demands, and family obligations. For some, scheduling difficulties complicated their ability to attend. Others emphasized creative problem-solving to ensure participation, such as rearranging radiation sessions or meetings. These narratives suggest that scheduling barriers were more about negotiating competing priorities, where attendance reinforced participants’ commitment to participating in golf.

##### 3.1.3.2 Subgroup #2: physical health barriers

For four participants, *physical health barriers*, including injuries, chronic conditions, and treatment-related fatigue, were described as both limitations and motivators for participation. Some talked about temporary setbacks, such as a groin injury or shoulder pain, that made participation more difficult. Others highlighted how health barriers prompted them to adapt their routines, such as separating physical therapy and golf sessions to maximize recovery. Rather than discouraging engagement, these health challenges often became opportunities for participants to demonstrate resilience and self-management. By working through pain and fatigue, they reinforced their sense of progress and control over their health.

##### 3.1.3.3 Subgroup #3: social barriers

One participant reflected on *social barriers*, where family crises or caregiving responsibilities occasionally pulled them away. This shows that when participants missed sessions, it was not due to a lack of interest or commitment, but because family and community obligations took priority. In many ways, these moments highlighted how important social ties were in their lives, and how participation had to be balanced alongside those responsibilities.

Overall, barriers like scheduling conflicts, health problems, or family issues did not stop people from staying connected. Instead, working through these challenges highlighted their determination to participate and the importance they placed on being part of the group and participating in golf.

#### 3.1.4 Theme 4: expectations and reflections

##### 3.1.4.1 Subgroup #1: continued engagement

Most participants expressed continued *intent to play golf* in some form after the program, though how they pictured doing so varied depending on their goals, social connections, and daily responsibilities. For many, golf had shifted from being an unfamiliar activity to something enjoyable and sustainable. Seven people spoke with certainty about their intent to keep playing, often emphasizing that golf provided the right balance of physical activity and leisure. These reflections suggest that participants were not just interested in continuing but were also actively shaping the way golf could fit into their lives long term.

For seven participants, continuing golf meant *increasing social engagement*. Several participants talked about playing with family members, such as children or grandchildren, or reaching out to neighbors and friends who also played golf. Some also reflected on choosing partners who matched their pace, suggesting that enjoyment came not just from the game itself but from the social context in which it was played. Others talked about keeping in touch with people they met in the program itself and planning to play together. For the individuals in the program, golf had become more than just an individual activity; it offers a new way of connecting socially and strengthening existing relationships.

All ten participants also expressed their *intent to play with location-based planning*. Some mentioned choosing courses close to home, noting that travel time would be a deciding factor in whether they kept up with the sport. Others focused on the physical challenge of certain courses, looking for flatter or less demanding terrain. These reflections show how decisions to continue are influenced by practical and logistical considerations.

Nine participants reported that they would *continue attending if the program itself were extended*. The structure, accountability, and group dynamic were central motivators. For these individuals, the organized format, fostering an environment of learning, support, and accountability, offered benefits beyond what self-directed play could provide.

Three participants imagined *continuing golf with a modified play format* to make golf more sustainable. Playing 9 holes instead of 18, sticking with the driving range, or using push carts instead of riding were all described as ways to adapt the activity to their health and lifestyle.

Altogether, participants’ reflections on continued engagement showed a mix of enthusiasm, practicality, and adaptability. The program helped participants envision how to integrate golf into their routines in ways that matched their physical abilities, social networks, and daily lives. For many, expectations went beyond simply playing more golf and included broader commitments to staying active, connected, and intentional about personal health.

##### 3.1.4.2 Subgroup #2: program expectations

Many participants came into the program with *initially unclear expectations*, many of which were shaped by uncertainty, curiosity, or skepticism. Six participants admitted they felt nervous before the first session, unsure if they belonged. For others, expectations were unclear. These reflections show how unfamiliar and novel the concept of the program felt for many participants, and how participation felt like both a risk and an opportunity.

At the same time, two participants entered with concrete *quality instruction expectations*, noting the credibility of a PGA-certified program and referrals from trusted sources. These participants framed their involvement as a chance to learn from experts in a structured, professional environment.

For many, expectations were tied less to technical golf skills and more to *holistic benefits*, particularly physical activity, emotional well-being, and social connection. Some highlighted the way the program motivated them to be more active beyond the sessions. For many, expectations reflected broader goals of recovery, health, and a journey toward building community.

Three participants also noted *unexpected benefits*, including the surprise of receiving free clubs and equipment. These unanticipated elements enhanced participants’ motivation and made the program feel more rewarding.

Across the board, participants emphasized that their *expectations were not only met but often exceeded*. What began with hesitation transformed into accomplishment, belonging, and enjoyment. This sense of exceeding expectations was echoed repeatedly, as participants spoke of discovering new confidence, improved health, and meaningful social ties that went far beyond what they had anticipated at the start.

Taken together, participants’ reflections reveal that while many entered the program with little clarity about expectations, the combination of professional instruction, holistic benefits, and unexpected opportunities created an overall transformative experience.

## 4 Discussion

Overall, the program met the hypotheses of feasibility with few minor program-related adverse events, a high adherence rate, high satisfaction, and high enjoyment. The quantitative results demonstrated tangible evidence of the program’s feasibility, whereas the qualitative results provided insights into the elements needed to optimize a multimodal program that is feasible, enjoyable, and adherent.

The quantitative variables measured included adherence rates, adverse events, PACES-8 enjoyment scale, and a 4-item satisfaction scale across the program duration. The session attendance rate was 90%, and the within-session adherence rate was 98.9%, which are among the highest rates in exercise intervention studies in men with PCa (average adherence: 80.38%; range 49% - 94%; **Table 2**) (9). There were just a few Grade 1 adverse events, which highlight the safety of the program in older-adult PCa survivors undergoing hormone treatment with minimal/no golf experience (21). Enjoyment and satisfaction of the activity are two theoretical constructs that relate to the intrinsic motivation of individuals to sustain long-term participation in physical activity (22, 23, 27). Recently, Naismith et al. explored exercise motivation in PCa survivors undergoing ADT through self-determination theory and found that enjoyment is a key intrinsic motive for activity engagement (27). Though recognized as important constructs for physical activity engagement and lifestyle modification, enjoyment and satisfaction are not often quantified and reported in PCa exercise intervention studies. PACES-8 is a validated instrument used to measure physical activity enjoyment in older adults and is frequently used in exergame intervention studies (24, 28, 29). Padala et al. conducted an 8-week Wii-Fit game-based exercise intervention with 15 older-adults and reported PACES-8 score of 49.2 ± 7.4 (28). Similarly, Bird et al. conducted a 5-week postural balance-focused exergame intervention in 24 older adults and reported PACES-8 score of 53 (SE: 0.7) (24). Comparatively, the current study found that PACES-8 scores were 53–55 across the study, indicating high enjoyment at each of the 3 phases of the training program. The findings are similar to the satisfaction scale, which is sensible because the two measures have previously been found to be correlated (r=0.72) (23). These quantitative measures coherently highlight the feasibility of the program.

In exercise oncology, there is an increasing awareness of the need to design exercise interventions that support psychosocial health and enjoyment aspects of the experience (11, 27, 30, 31). The qualitative analysis of the exit interview provided perspectives on the important factors to consider when designing participant-centered MRAs. As the results of thematic analysis describe, participants valued an overall enjoyable experience which stemmed from a well-designed program and structure, high quality and expert instructions, and social support from peers and instructors. These results indicate that the participants enjoyed the program not only because of physical exercise alone but also because of the psychosocial support the program provided. As demonstrated by the qualitative results, the participants enjoyed the social and skill-building aspects of golf and viewed the physical exercise component of the program as complementary to their golf skill learning. The research team emphasized delivering an enjoyable experience rather than making exercise or *golf performance* the focus of the program, which was well received by the participants. Thus, exercise alone should probably not be the sole focus of exercise oncology programs. For programs to be impactful, the design and execution should include holistic considerations that also emphasize participant social interaction and camaraderie. By their nature, *recreational* sports and activities implicitly incorporate many of these psychosocial benefits; thus, MRAs seem worthy of future study.

The barriers to participation were family/work obligations, medical appointments, unrelated injuries, and symptom management (e.g., fatigue), which are aligned with commonly reported barriers (7, 32). The program was structured, group-based, and outdoors. While such design factors were reported as top facilitators to physical activity participation, they come with challenges. The 5-person training group design required coordination of participants’ schedules. Most participants were retirees but still had family, work obligations, and medical appointments that prevented them from attending all sessions. Interestingly, 5 participants had to commute over 20 miles each way to come to the program facility, but travel distance was not reported as a prominent barrier to participation, despite the potential long traffic delays in the greater Los Angeles area. In this case, the participants seemed to value the facilitators of the program more than the hassle of a long commute. The average age of study participants was 73; the advanced age and treatment-related symptoms made them vulnerable to unrelated injuries and urinary incontinence. Future studies should incorporate more educational components about musculoskeletal injuries and how to manage physical demands outside of the structured programs to minimize injury risk. The study included pelvic floor muscle training as an emphasis of the program, which was reported as a helpful component by several participants. Lastly, cancer-related fatigue is a debilitating symptom but can be managed with exercise. PCa survivors should be educated about the benefits of exercise on managing fatigue and encouraged to continue participating to the best of their ability (10, 33, 34). Though not all barriers to participation can be overcome, future studies should incorporate the known facilitators such as structured, group-based programs with a variety of exercise/activity types to create a fun and social exercise experience to motivate the participants and increase adherence (7, 27, 32).

There were several limitations to the study. First, the within-subjects design required each participant to attend three data collection visits and wait 6 weeks from study enrollment due to the delayed-entry control period. The within-subject, delayed-entry design was chosen in accordance with the feasibility study recommendations by the ORBIT model for developing behavioral treatments for chronic diseases as well as limitation in available research resources (e.g., budget, time, staff) (18–20, 35). This study design theoretically allowed all enrolled participants the opportunities to receive the intervention and act as their own controls. Unfortunately, four participants dropped out during the control period due to unrelated injuries, disease progression, and loss of motivation (**Table 1**). Despite strong desires to continue with the study, the two participants who sustained unrelated injuries and the participant who experienced disease/symptom progression during the control were unable to safely complete the second data collection and the training program. The number of dropouts during the control period was unexpected but we believe it was related to the study design rather than the training program itself. There was only one dropout during week-6 of the training program, where a participant experienced worsened hip and back pain from an existing chronic condition. The study design did not allow new participants to be enrolled to replace the discontinued participants. The dropout pattern may have also affected interpretation of adherence. Attendance and within-session adherence were high among participants who initiated the intervention, indicating strong feasibility among those who began the active program. However, attrition during the delayed-entry control period may have resulted in a more motivated and physically able group entering the intervention, potentially overestimating adherence and acceptability. Future studies should consider shortening or eliminating the delayed-entry period, providing pre-intervention engagement during wait periods, or using alternative controlled designs to reduce early attrition while preserving methodological rigor. Secondly, due to the rise in popularity of golf post-COVID era, the golf course occasionally had high traffic which limited the pace of play on course. Another limitation is that the provision of free golf clubs and equipment may have acted as an external motivational factor that positively influenced participants’ perceptions of the program. Several participants specifically identified receiving equipment as an unexpected benefit, suggesting that this component may have contributed to their enjoyment, satisfaction, and willingness to continue participation. Therefore, the high PACES-8 and satisfaction scores may reflect not only the acceptability of the GREENS program itself, but also the added motivational value of receiving golf-related resources. Future studies should consider whether equipment provision is part of the intervention model or should be controlled for when evaluating acceptability outcomes. Lastly, the study was funded by generous individuals and organizations through a USC-sanctioned crowdfunding project. The budgetary limitation dictated that only a small cohort of participants could receive the intervention, though the sample size is not uncommon in feasibility studies of a novel intervention (11, 17, 28). Due to the small intervention-completer sample (N = 10), the findings should be interpreted primarily in relation to operational feasibility and acceptability. The high attendance rate, high within-session adherence, absence of Grade 2 or higher adverse events, and strong enjoyment and satisfaction scores suggest that the GREENS program was feasible to deliver and acceptable to participants who initiated the intervention. Similarly, qualitative findings provide insight into participants’ perceived experiences, including the value of social support, structured instruction, skill development, and program organization as factors that supported engagement.

## 5 Conclusion

In conclusion, the study demonstrated that the *GREENS* program was feasible with high safety, adherence, satisfaction, and enjoyment. It provides evidence that the multimodal intervention approach was popular among participants. Future research and use of golf-based and other MRAs in exercise oncology settings are warranted.

## Data Availability

The raw data supporting the conclusions of this article will be made available by the authors, without undue reservation.
