# Supplementary material for: Feasibility of the Golf Recreational Exercise for Enhanced Survivorship in prostate cancer survivors undergoing hormone therapy
Source: Front Oncol. 2026 Aug 11;16:1844481. doi: 10.3389/fonc.2026.1844481 (PMC13503178; doi:10.3389/fonc.2026.1844481)
Supplement: Supplementary file 1 [file DataSheet1.pdf]

Supplementary Material

Table 3. Qualitative Results and Associated Exemplar Quotes

Themes & Subgroups	Exemplar quotes from each category
**Themes 1: Perceived Value & Satisfaction**	
**Overall Satisfaction**	***Holistic health benefits**: "The program helped a lot because I can keep myself moving, keep myself thinking about what I am going to do, and getting better." (G11)*<br><br>*"Because it helps me mentally. It skips the constant thought of cancer. It keeps you busy, physically active. I like the exercises we do before golf." (G13)*<br><br>*"I wanted to improve my health, agility, concentration—all of that was important. I didn't want to miss out on that." (G14)*<br><br>***Social and emotional support**: "It is not just about golf, it's about community, sharing our cancer journeys, and staying optimistic. It has far-reaching consequences, including just feeling good about where we are in our station in life." (G02)*<br><br>***Learning and self-growth**: "learning golf is something I've never done before and recognizing it's as complex and rich as learning to swim, with all the different elements needing to be learned, understood, and integrated... was very exciting." (G01)*<br><br>*"...it improves physical and emotional well-being and helps you reflect on your own situation. It makes you feel less sorry for yourself when you hear what others are going through" (G16)*<br><br>***Program recommendation:** "Seven (indicated "totally will recommend") — definitely. Absolutely, I would. This is a great program. I'm glad I joined. It helps you physically, mentally, and socially." (G17)*<br><br>*"The whole program was a 10 out of 10." (G05)*

**Structure and Format**	***Appreciation for structure and organization:*** *"I really wouldn't change anything. It's a well-laid-out program—the exercises, the golf lessons, and getting out on a course—it's all great." (G05)*<br><br>*"I liked my researcher, my instructor, I liked the guys I golfed with, I liked the facility—it had everything we needed. You (the research coordinator) and Kevin know what you guys are doing." (G01)*<br><br>***Desire for more golf time:*** *"Obviously, more golf is better. But the exercises were helpful, they made me more flexible and taught me good warm-up exercises I can use after the program." (G10)*<br><br>***Desire for more personalize feedback*** *"The instruction from Kevin could be improved a little bit, with a little more personal touch." (G13)*<br><br>***Satisfaction with the pre-golf exercises:*** "The exercises were great—they targeted the areas I needed most, especially my urination. And golf turned out to be a lot more fun than I expected." (G16)<br><br>***Preferences for logistical changes:*** *"Maybe just the schedule—I'd prefer earlier in the day instead of around noon… Plus, I think the duration was right. The 10-week structure with 20 sessions was wonderful." (G01)*
**Theme 2: Program Design & Delivery**	
**Instruction Quality**	***Positive and supportive teaching style:*** *"And Kevin, was just exquisitely just a capable instructor. He does all the right things—he gives you a small cue, encourages you when you do it right, and keeps things positive." (G01)*<br>*"Honestly—had it been different personalities, who knows. Kevin made it really accessible. You made it really accessible. I didn't feel stressed about competition." (G07)*<br><br>***Desire for increase details:*** *"Yeah. Just more detail—especially when it came to grip and angulation. He covered the fundamentals, but the points he made were really valid… all of that is really important." (G02)*<br><br>***Clarity and effectiveness of instructions:*** *"He gives just the right amount of instruction. Minimal but to the point" (G01).*<br><br>*"[The instructor was] very calm… laid-back but clear, not overly strict. He was very good at taking you where you are and gave you very broad instructions on how to improve" (G10).*
**Engagement and Motivation**	***Sense of belonging:*** "It was a good association with the patients that were there—our guys—and with you and Kevin. I found a lot of support for an

	activity I hadn't done in years and years… you put us all at ease straight away" (G07).<br><br>"The fun and interaction with the other guys, plus the thought of playing golf and hitting the ball—even just at the driving range—and the exercise too" (G16).<br><br>***Access to resources:*** *"The good friendships. Free golf clubs, free golf sessions, and being more physically active. Meeting more people. It was very motivating… it seemed too good to be true." (G17)*<br><br>***Instructor encouragement:*** *"You, as the program director, were very encouraging." (G10)*<br><br>***Connection with golf:*** ***"****Two things—learning golf and wanting to know how I was doing physically with my condition… I'd say, 'This is great, but this part doesn't seem to be working,' and I'd be like, 'That's weird." (G13)*<br><br>*"I used to like to play golf before… I'm coming back, I'm going to play again." (G11)*
**Theme 3: Challenges and Barriers**	
**Time and Scheduling**	"I always looked forward to coming out here, learning more about golf, and doing the exercises. I knew it would benefit me. I think I only missed one session, and that was because of a doctor's appointment for a relative." (G05)<br><br>"I had some scheduled meetings I couldn't postpone, but luckily I was able to schedule radiation early in the morning so I could still attend." (G10)
**Physical Health Barriers**	*"At first, it was my shoulder pain from my dog pulling me. It made it hard to raise my arm. But with the exercises, it improved a lot—about 95% better now." (G16)*<br><br>"At first, I scheduled PT and golf on the same day, but I learned quickly and changed my schedule so I could recover. Now I do not do PT and golf on the same days anymore. I do them on different days." (G17)
**Social or Emotional Barriers**	"The only barriers I had were family issues. Friends and family stuff. I missed one because [my wife] had her ankle surgery. And our friend's house burned down—I wasn't going to leave them." (G07)
**Theme 4: Expectations and Reflections**	
**Continued Engagement**	***Intent to play golf:*** "Very likely—definitely a 7. I would love to continue. I'm looking forward to improving my game. It's a big challenge and one I enjoy." (G05)

	Increasing social engagement: "Yes, I'll play with my grandson and my son—possibly at Pico Rivera." (G14)<br><br>"I plan to play with family or friends I met in the program. I definitely want to keep playing. I'd never played golf before, and I really enjoyed it, even at age 70" (G17).<br><br>***Intent to golf with location-based planning:*** "If I play— 'perhaps' is as strong as I can put that—I'll play at Arroyo, because it's closest to my house" (G07).<br><br>"Maybe somewhere easier than Monterey Park. That one has a lot of hills, and it's tough for me to go up and down." (G17)<br><br>***Continued engagement if program was extended:*** "Oh, I would continue to come to the program as long as it lasted." (G07)<br><br>"Very likely—definitely a 7. I would love to continue. I'm looking forward to improving my game." (G05)<br><br>***Intent to golf with modified formats:*** "I prefer playing 9 holes—it feels like the perfect distance for me right now. I don't really want to use a cart. I want to carry my clubs or use my pushcart, and that's part of my physical conditioning." (G02)
**Program Expectations**	***Initially unclear expectations:*** *"I had zero expectations. I had no idea what I was getting into. I was afraid of it. I was apprehensive the night before, thinking maybe I shouldn't have signed up, because I knew I'd suck at golf." (G07)*<br><br>*"I didn't really know what to expect when I started, but once I got into a rhythm, I could concentrate more." (G13)*<br><br>***Quality instruction expectations:*** *"I expected quality instruction since it was a PGA-certified program. From what I knew about you, and what Mary had told me, I felt confident that it would be something I'd enjoy and get involved in. So my expectations were high and expectations were absolutely met completely and then some." (G01)*<br><br>***Holistic benefits expectations:*** "My expectation was to learn golf and improve my physical ability, and to connect with others suffering from the same disease. Those expectations have definitely been met." (G10)<br><br>"It's helped me stay motivated to continue exercising, to get out and walk, and to go to the golf course and swing that club." (G05)<br><br>***Unexpected benefits:*** *"Everyone was supportive. I was apprehensive at first, but even though Alex had a full bag of clubs and covers—you could be intimidated—*

you guys gave us clubs, which was a surprise. I thought they were rentals." (G07)

Expectations exceeded: *"Oh yeah. Not only met but exceeded. At first, I thought, 'Yeah right, you're going to teach me golf,' and I was skeptical. But I wanted to learn golf and was curious how it would go. It turned out really nice. Actually, I don't just go—I look forward to Mondays and Wednesdays now." (G13)*

"But the program definitely exceeded any expectations I could have had. I didn't know what to think at first—but it turned out to be so much more." (G02)
